# The cellular prion protein is a stress protein secreted by renal tubular cells and a urinary marker of kidney injury

**DOI:** 10.1038/s41419-020-2430-3

**Published:** 2020-04-17

**Authors:** Yohan Bignon, Virginie Poindessous, Hélène Lazareth, Bruno Passet, Jean-Luc Vilotte, Fatima Djouadi, Sophie Mouillet-Richard, Nicolas Pallet

**Affiliations:** 1 INSERM U1138, Centre de Recherche des Cordeliers, INSERM, Sorbonne Université de Paris, F-75006 Paris, France; 2 Service de Néphrologie, Hôpital Européen Georges Pompidou, Assistance Publique Hôpitaux de Paris, Paris, France; 3 Université Paris Saclay, INRAE, AgroparisTech, UMR1313, Génétique animale et biologie intégrative, F78350 Jouy-en-Josas, France; 4 Service de Biochimie, Hôpital Européen Georges Pompidou, Assistance Publique Hôpitaux de Paris, Paris, France

**Keywords:** Prions, Diagnostic markers

## Abstract

Endoplasmic Reticulum (ER) stress underlies the pathogenesis of numerous kidney diseases. A better care of patients with kidney disease involves the identification and validation of ER stress biomarkers in the early stages of kidney disease. For the first time to our knowledge, we demonstrate that the prion protein PrP^C^ is secreted in a conventional manner by ER-stressed renal epithelial cell under the control of the transcription factor x-box binding protein 1 (XBP1) and can serve as a sensitive urinary biomarker for detecting tubular ER stress. Urinary PrP^C^ elevation occurs in patients with chronic kidney disease. In addition, in patients undergoing cardiac surgery, detectable urine levels of PrP^C^ significantly increase after cardiopulmonary bypass, a condition associated with activation of the IRE1-XBP1 pathway in the kidney. In conclusion, our study has identified PrP^C^ as a novel urinary ER stress biomarker with potential utility in early diagnosis of ongoing acute or chronic kidney injury.

## Introduction

Kidneys have to cope with a wide array of injuries that translate into elementary stressors at the cellular level. Adaptive responses to these stresses are molecular systems that primarily aim to eradicate or reduce stress intensity and promote metabolic reprograming to maintain cellular homeostasis and other vital functions^1^. Adaptive stress responses shape the endogenous repair and scarring equilibrium in tissues. As such, they are critical for tissue remodeling, and can impact the functional outcomes of the injured kidney, ultimately leading to chronic kidney disease (CKD)^2,3^. The molecular reprogramming circuitries and cellular adaptive responses that occur in renal epithelial cells early after the initiation of injury, before cell death and the engagement of the maladaptive repair process, offer the opportunity for a non-invasive monitoring and the implementation of potential nephroprotective strategies. For this reason, the characterization of the “secretome” produced by tubular cells is critical for the design of urine markers of kidney injury.

Endoplasmic reticulum (ER) stress and its adaptive response, the unfolded protein response (UPR), are archetypal examples of these adaptive stress responses, and the detection of its activation constitutes an opportunity for an early diagnosis of ongoing tissue injury. Of particular interest in the UPR is the IRE1α-XBP1 axis. IRE1α catalyzes the unconventional processing of the mRNA encoding the transcriptional factor X-Box binding protein-1 (XBP1) and creates a transcriptionally active XBP1 (sXBP1) that contains a potent transactivation domain and enters the nucleus. Genes that are regulated by sXBP1 enhance protein folding, transport, and degradation, as well as the expansion of the protein secretory pathways. XBP1 is critical for cellular adaptation in stressful conditions. Whether sXBP1 activity is specifically implicated in the pathophysiology of kidney disease is mostly unknown, but recent studies have indicated that sXBP1 is required for podocytes, mesangial and tubular cells maintenance under stressful conditions^4–7^, or, on the contrary, contributes to renal inflammation and injury^8^. The clinical consequence of the activation of this pathway would be that the detection of sXBP1 or its target genes may have prognostic value. Since XBP1 splicing depends exclusively on IRE1α activity, sXBP1 is only produced if and when ER stress occurs (with a rare exception), therefore reflecting the presence of ER stress with high sensitivity.

Although multiple lines of evidence from clinical and experimental studies have demonstrated that a maladaptive ER response is mechanistically linked to the pathogenesis of kidney diseases, a lack of robust biomarkers for monitoring renal ER stress has hampered early detection and effective therapeutic intervention to restore ER homeostasis. Here, we investigated if the soluble form of the ER stress-inducible protein, prion protein (PrP^C^) is also a candidate urinary ER stress biomarker. The cellular prion protein PrP^C^, which is involved in the pathophysiology of some neurodegenerative diseases under its pathogenic scrapie isoform, is a ubiquitous protein mainly found as a GPI-anchored cell surface molecule and present in the extracellular space as a soluble isoform^9^. The aim of this study was to determine whether PrP^C^ could serve as a urinary ER stress biomarker for ER stress-mediated kidney diseases by studying a combination of cellular models and human patient samples.

## Material and methods

### Reagents

Tunicamycin (Tun), Thapsigargin (Tg), Brefeldin A (BFA), Etoposide (Eto), DL-Dithiothreitol (DTT) and mouse monoclonal antibody against α-tubulin were from Sigma Aldrich (St. Louis, MO, USA). Mouse monoclonal antibodies against PrP^C^ (Sha31) were from SPI-Bio (Montigny Le Bretonneux, France). Goat polyclonal antibodies against BIP (GRP78) were from Santa Cruz (SC-1050 Santa Cruz, CA, USA). Polyclonal rabbit antibodies against PARP (#9542) and PERK (#3192) were from Cell Signalling Technology (Danvers, MA, USA).

### Cell culture and treatments

Human Renal Epithelial Cells (HREC) of proximal origin immortalized with the HPF 16 E6/E7 genes (HK-2) were purchased from American Type Culture Collection (lot #710257641). Primary HREC were harvested from a human nephrectomy specimen. This cellular model has been previously molecularly characterized confirming the proximal descent of the vast majority of the cultured tubular epithelial cells^10^. HREC were cultured at 37 °C in an atmosphere containing 5% CO_2_ in Dulbecco’s Modified Eagle’s Medium (41965–039 Gibco, MD, USA) supplemented with 1% fetal bovine serum (HyClone, SV30160.03 GE lifesciences), 0.5 µg/ml hydrocortisone (Sigma-Aldrich), 1X Insulin Transferrin Selenium (Sigma-Aldrich), 10 ng/mL Epithelial Growth Factor (Sigma-Aldrich), 6.5 ng/mL Triiodothyronine (Sigma-Aldrich), and 1X of Penicillin-Streptomycin mix (Gibco). Mycoplasma-free cells (Mycoalert Mycoplasma Detection Kit, Lonza) were subcultured before complete confluence using 0.05% Trypsine-EDTA (Gibco) and experiments were not performed with cells beyond the third passage since it has been shown that no phenotypic changes occur up to this passage number.

Sub-confluent cells were grown in six-wells plates for the indicated times under the indicated conditions. Unless otherwise stated, Tun was used at 250 ng/ml, Tg at 250 nM, DTT at 1 µM, BFA at 5 µg/ml and Etoposide at 100 µM. For glucose deprivation, cells were grown during 48 hours in glucose free Dulbecco’s Modified Eagle’s Medium (11966–025 Gibco) with control cells supplemented with 4.5 g/L glucose (Gibco). Cell culture media were collected and cleared through centrifugation at 5000 g during 5 minutes then culture supernatants were immediately stored at −80 °C prior analysis. For transient siRNA-mediated silencing, cells were transfected with siRNA sequences (50 nM) using the Lipofectamine 2000 reagent according to the manufacturer’s instructions (Invitrogen, Carlsbad, CA, USA). Specific siRNA sequences used were: 5′-CAGUACAGCAACCAGAACATT-3′ (sense si-PRNP). siRNA against XBP1 (Hs_XBP1_7 and Hs_XBP1_7) was from Qiagen. AllStars Negative Control siRNA (5′-AACGAUGACACGAACACACTT-3′) has no homology to any known mammalian gene, and validation has been performed using Affymetrix GeneChip arrays and a variety of cell-based assays to ensure minimal nonspecific effects on gene expression and phenotype.

### Cell proliferation and viability assays

For cell counting experiments, 2.5 × 10^5^ cells were seeded in six-well plates and transfected with siRNA against *PRNP* 24 h post seeding. After another 24 h, culture medium was replaced by fresh medium containing siRNA and Tun or Tg. After 24 h of incubation, cells from three wells per group were harvested and total cell numbers (cells/well) were determined using a CASY.TT cell counter (Schärfe System GmbH, Reutlingen, Germany). Cells transfected with control siRNA and treated with vehicle (DMSO) were used as reference for normalization.

### Immunoprecipitation

Immunoprecipitation of PrP^C^ was performed using Dynabeads Protein G magnetic beads (Life technology), according to manufacturer’s protocol. Briefly, 50 µL of washed beads were incubated with 1 µg of anti-PrP^C^ antibody under rotation for 1 h at 4 °C. Crosslinking of antibody to the beads was performed with the BS3 (bis(sulfosuccinimidyl)suberate) linker (Thermofischer). Immunoprecipitation was performed with 250 µL of culture supernatant of HK-2 cells overnight under rotation at 4 °C. The immuno-precipitated material was eluted in elution buffer and analyzed by western blot.

### Protein extraction and western blot analysis

Cells were washed in PBS and incubated for 30 min at 4 °C in NaDOC lysis buffer [50 mM Tris·HCl (pH 7.4)/150 mM NaCl/5 mM EDTA/0.5% Triton X-100/0.5% sodium deoxycholate] and a mixture of phosphatase (Thermo-Scientific, Waltham, MA, USA) and protease (Roche, Mannheim, Germany) inhibitors. Extracts were centrifuged at 14,000 g for 15 min and protein concentration in supernatants were measured using the bicinchoninic acid method (Pierce, Rockford, IL, USA). Deglycosylation was performed on 15 µg of proteins with 500U PNGaseF (New England Biolabs, Ipswich, MA, USA) for 1 hour at 37 °C. In all, 25 µg of protein extracts were resolved by 4–12% SDS-PAGE (Invitrogen) and transferred to nitrocellulose membranes (iBlot, Invitrogen). Membranes were blocked with SEABLOCK blocking buffer (Thermo-Scientific) for 1 h at room temperature and then incubated overnight at 4 °C with primary antibody diluted in blocking buffer. After washings in PBS-Tween buffer, membranes were incubated with secondary antibody coupled to IRDye fluorophores. Infrared signal of membranes was revealed using an Odyssey detection system (Li-Cor biosciences, Lincoln, NE, USA).

### RNA extraction and real-time qPCR analysis

Total RNA was extracted using the RNeasy Mini Kit® (Qiagen) following the manufacturer’s protocol. The yield and purity of RNA were measured using a NanoDrop ND-1000® spectrophotometer (Nanodrop Technologies). For reverse transcriptase-polymerase chain reaction (RT-PCR) analysis, first-strand cDNA was synthesized on 1 µg of RNA with oligo(dT) primer and random 6mers, using the High-capacity cDNA Reverse Transcription (Applied Biosystems) according to the manufacturer’s protocol. Real-time PCR was performed using Absolute QPCR SYBR Green ROX Mix (Thermo-Scientific, Waltham, MA, USA) on a ABI PRISM 7900HT (Applied Biosystems, Life Technologies Corporation, Carlsbad, CA, USA). Real-time PCR analyzes were performed with the SDS software 2.3 (Applied biosystems). Primers used for the PCR reactions are shown on Supplementary Table 1. Results are expressed as a relative quantification of a target gene transcript normalized to the *RPL13A* (human samples) or *Tbp* (mouse samples) housekeeping gene using the ΔΔCt method.

### Enzyme-linked immunosorbent assays

Soluble PrP^C^ was quantified in cell culture supernatant or urinary samples using the BetaPrion Human ELISA immunoassay (Analytikjena Leipzig, Germany), according to the manufacturer’s protocol. Soluble Angiogenin and neutrophil gelatinase-associated lipocalin (NGAL) were quantified in urinary samples using the Quantikine® human ANG and NGAL immunoassays (RD Systems), according to the manufacturer’s protocol.

### Animal studies

Analyses were carried out on kidneys from 4 month-old-male C57BL/6 mice exposed to one injection of Tunicamycin (intraperitoneal injection, 1 mg/kg) or Vehicle (DMSO) and collected 48 h after injection^11^. Samples were analysed under blinded conditions.

### Human studies

#### Urinary PrP^C^ concentration measurements in individuals with Chronic Kidney Disease

In all, 55 consecutive patients who were referred to the Nephrology Department at the Georges Pompidou European Hospital (Paris, France) for kidney biopsy from 7 December 2017 to 22 February 2018 were included. Indications for biopsy were estimated GFR < 60 ml/min and/or proteinuria >0.5 g/L. Kidney biopsies were not performed for the purpose of this non-interventional study, but only for patient care. At the time of biopsy, urine samples were collected for routine clinical chemistry analyses and stored at −80 °C. Samples were analyzed under blinded conditions. Patients provided informed consent that their urine specimen could be used for research purpose.

#### Urinary PrP^C^ concentration measurements in individuals with cardiopulmonary bypass

To monitor PrP^C^ in the setting of ischemia-reperfusion injury (IRI), we took advantage of the biocollection of a previous study^12^. From 17 February 2017 to 26 April 2017, 42 patients undergoing scheduled cardiac surgery with CPB were enrolled. The exclusion criteria were: an eGFR <30 ml/min/1.73 m^2^, infusion of a radio contrast agent within the 24 h before surgery, a preoperative left ventricular ejection fraction <40%, age <18 years, pregnancy, and the inability to provide consent. AKI was diagnosed according to the KDIGO Clinical Practice Guideline for AKI criteria (http://kdigo.org/) using serum creatinine levels and urine output after the surgery. This single-center, prospective, pilot study was approved by the French ethical committee on 7 February 2017 (CPP Sud Est III n° 2016–072 B) and registered under the EudraCT n° 2016-A01871–50. All patients provided written consent for study participation and for the biological analysis before inclusion. Urine samples were collected from a urinary catheter at three different times: (1) after the induction of anesthesia and before the start of cardiopulmonary bypass (CBP), (2) at the end of the CPB procedure, and (3) on the day after surgery in the ICU. Urine samples were collected in Corning 50-ml conical tubes and centrifuged at 2000 *g* for 20 min within 4 hours of collection. Cell pellets were conserved in 350 μL of RLT® buffer (Qiagen™, France) and stored until mRNA extraction. Supernatants and cell pellets were stored at −80 °C until analysis. Clinical data were prospectively extracted from the hospital’s electronic medical records. All clinical data and samples were de-identified. Urine PrP^C^ monitoring has been performed in a subgroup of 19 patients in whom urine samples were available at the three time-points for PCR and ELISA analyses. Experiments were all carried out under blinded conditions.

#### Immunohistochemistry of human kidney biopsies with chronic kidney disease

Three kidney biopsies from patients explored for chronic kidney disease were retrospectively analyzed for PrP^C^ immunohistochemistry studies. Kidney biopsies were not performed for the purpose of this non-interventional study, but only for patient care. Patients provided informed consent that these kidney biopsy specimen could be used for research purpose.

### Fragment analysis

RNA was extracted from the pellets of urinary cells using a RNeasy Mini Kit® (Qiagen) and reverse-transcribed into cDNAs using TaqMan® Reverse Transcription Reagents (Applied Biosystems). Basically, the fragment analysis involved the following 3 steps: (1) Amplification of the sXBP1 and XBP1 cDNAs by PCR using fluorogenic oligonucleotide primers. Several differently colored fluorescent dyes are detectable in one sample. We designed the following fluorogenic oligonucleotide primers (tagged with hexachloro-fluorescein, HEX, green) for fluorometric detection of the sXBP1 and XBP1 mRNA levels: forward primer: (5′-HEX)GGAGTTAAGACAGCGCTTGG-3′ and reverse primer: 5′-GAGATGTTCTGGAGGGGTGA-3′. We performed PCR using HotStart Taq® DNA polymerase on a thermal cycler with the following program: 95 °C for 10 min; 40 cycles of 94 °C for 30 s, 59 °C for 30 s, and 72 °C for 30 s; and a final step of 72 °C for 10 min. (2) Labeled fragments (amplicons) were separated by size using capillary electrophoresis, and the fluorescence intensity was measured using the Applied Biosystems™ 3730xl DNA Analyzer. One of the dye colors (GENESCAN® ROX 400 HD size standard, Applied Biosystems™, red) was used to detect a labeled size standard in each sample. Fragments and ROX 400 HD were mixed with HiDi™ Formamide (Applied Biosystems) prior to capillary electrophoresis. (3) The data were analyzed using GeneMapper® Software to determine the relative size of each dye‐labeled fragment in the sample by comparing fragments with the standard curve for that specific sample.

### Immunohistochemistry

Kidney biopsies were fixed in alcohol-formalin-acetic acid, dehydrated with ethanol and xylene, embedded in paraffin, and cut into 3 μm sections. Samples were then deparaffinized, rehydrated and heated for 20 min at 97 °C in citrate buffer. Endogenous peroxidase was inactivated by incubation for 10 min at room temperature in 0.3% H_2_O_2_. Sections were incubated with PBS containing 1:1000 anti-PrP^C^ antibody (Sha31, SPI-Bio) or control isotype (mouse IgG1κ, Biolegend, San Diego, CA, USA). Next, sections were incubated with anti-rabbit conjugated with peroxydase labeled polymer (Dako), visualized with a peroxydase kit (Dako). Finally, the tissue sections were counterstained with hematoxylin.

### Statistical analysis

All data are represented as individual values and means ± SEM of at least two independent experiments, unless otherwise specified. Graphs were generated using GraphPad Prism 7 Software (GraphPad Software, Inc.). According to current discussion and criticisms on the performance and interpretation of statistical tests in experimental settings, and in particular the misuse and misinterpretation of *p* values^13–15^, we choose not to systematically perform comparison of biological data using statistic tests to compute *p* values for significance. When deemed necessary, ie when the size effect appeared small, we provided mean and 95% confidence intervals of the two groups of values for interpretation.

## Results

### ER stressors promote *PRNP* mRNA and PrP^C^ protein expression in HREC

**Fig. 1:**
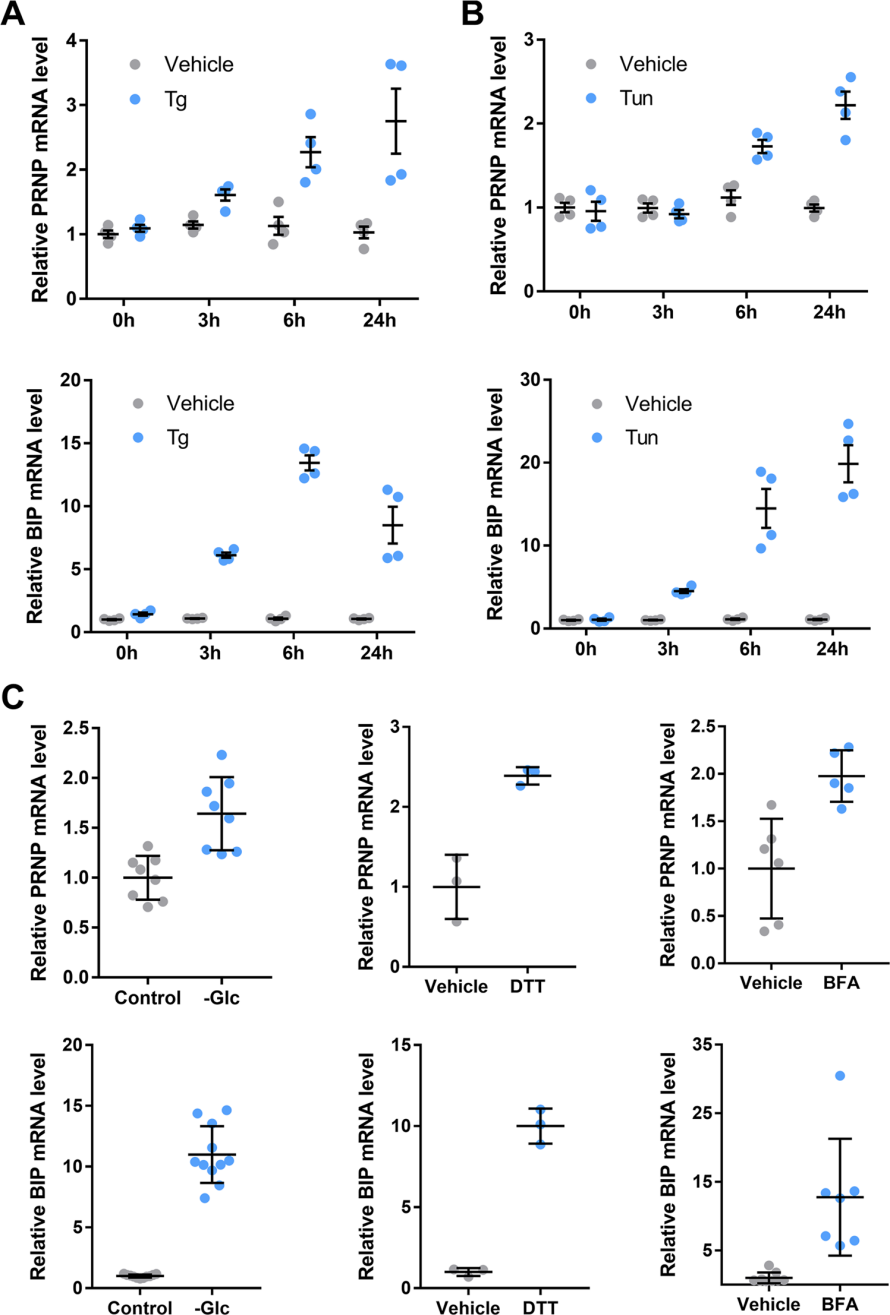
*PRNP* expression is induced by ER stressors. **a**, **b** Kinetics analysis of *PRNP* (top panels) or *BIP* (bottom panels) mRNA expression in HREC exposed to Tg (**a**) or Tun (**b**) compared to vehicle-treated cells. **c** qRT-PCR analysis of *PRNP* (top panels) or *BIP* (bottom panels) mRNA expression in HREC submitted to glucose deprivation for 48 h (left panel) incubated with DTT (middle panel) or BFA (right panel) for 24 h. Results are represented as individual values with means of *n* = 2–3 independent duplicates or triplicates of cell preparations ± SEM.

**Fig. 2:**
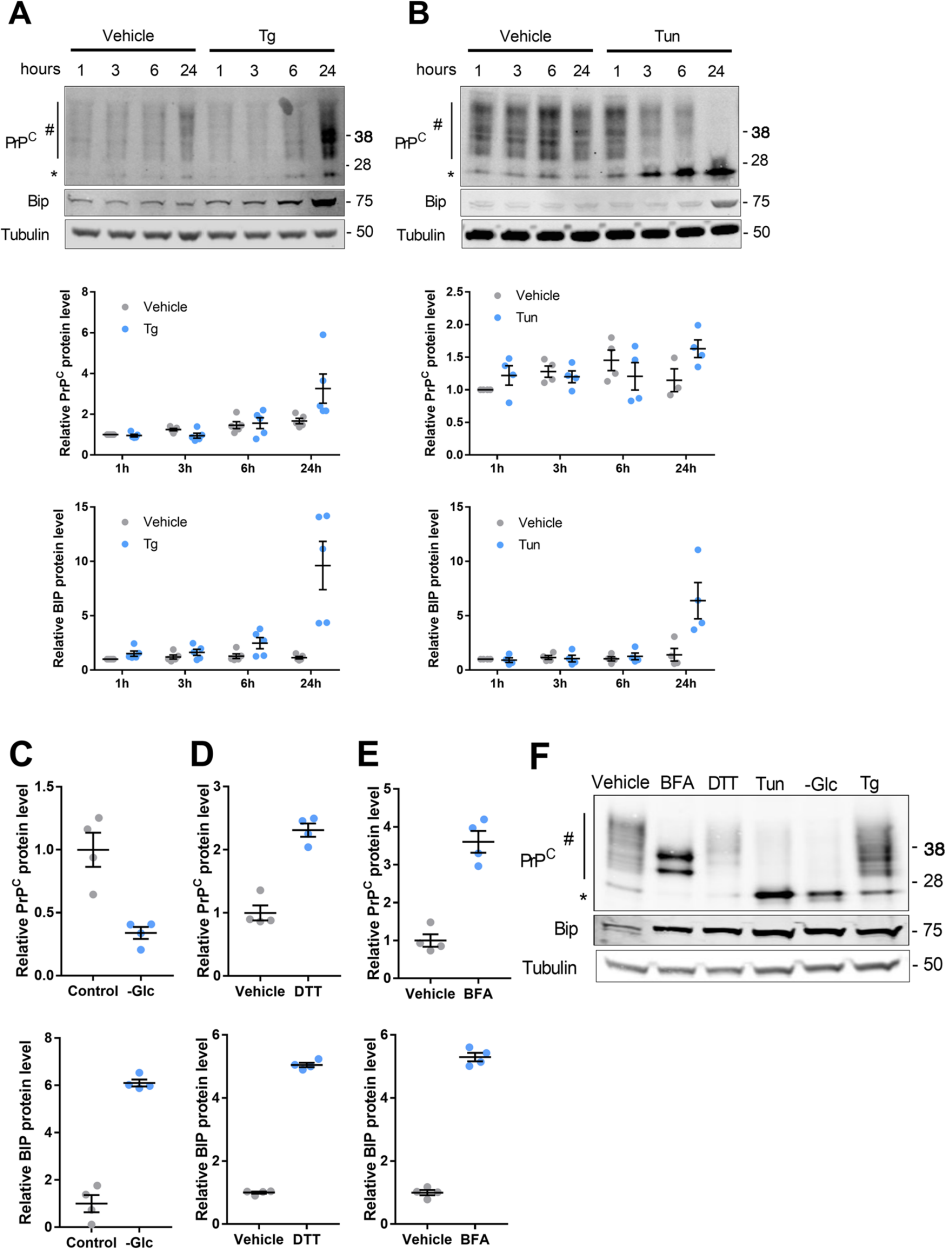
Impact of ER stressors on PrP^C^ protein expression. **a**, **b** Representative images and kinetics analysis of PrP^C^ (top panels) or BIP (bottom panels) protein expression in HREC exposed to Tg (**a**) or Tun (**b**) compared to vehicle-treated cells. **c**–**e** Protein quantification of PrP^C^ (top panels) or BIP (bottom panels) protein expression in HREC submitted to glucose deprivation for 48 h (**c**), incubated with DTT (**d**) or BFA (**e**) for 24 h or (**e**). Protein levels were normalized to Tubulin. Corresponding Western blot are displayed in Supplementary Fig. 2. **f** Comparative migration profile of PrP^C^ isoforms in HREC according to ER stressors. *Hash* indicates mono- or bi-glycosylated PrP^C^ isoforms. *Asterisk* indicates unglycosylated PrP^C^. Results are represented as individual values with means of *n* = 2–3 independent duplicates or triplicates of cell preparations ± SEM.

To assess whether the PrP^C^-encoding *PRNP* transcripts are sensitive to ER stressors in human renal epithelium, we monitored *PRNP* mRNA expression during a 24-h kinetics of exposure of Human Renal Epithelial Cells (HREC) to 250 nM Thapsigargin (Tg) or 250 ng/ml Tunicamycin (Tun). As shown in Fig. 1a, b, both treatments induced *PRNP* (top panels) and *BIP* (bottom panels) transcripts expression as measured through RT-qPCR. *PRNP* induction was mild after 3 h for Tg and induction started after 6 h for Tun. Twenty-four hours after ER Stress induction, *PRNP* expression reached over 200% of its basal levels. As depicted in Fig. 1c, such increase in *PRNP* (top panels) and *BIP* (bottom panels) transcription extended to ER stress resulting from a 48-hour glucose deprivation (-Glc), as well as a 24-h treatment with 1 mM of Dithiothreitol (DTT) or 5 µg/ml of Brefeldin-A (BFA) (Fig. 1c, bottom panels). Likewise, robust inductions of UPR transcripts and *PRNP* mRNA were found in primary HREC exposed for 24 h to Tg, Tun or BFA (Supplementary Fig. 1A). Corroborating these data, we observed an upregulation of *PRNP* mRNA together with UPR transcripts in the kidneys of mice collected 48 h after an intraperitoneal injection of Tunicamycin (1 mg/kg) (Supplementary Fig. 2), an established experimental animal model of ER stress^11^. At the protein level, a 24-h Tg treatment induced a robust increase in PrP^C^ expression in HREC (Fig. 2a), in line with the kinetics of *PRNP* mRNA induction previously described. With Tun, which inhibits the N-linked glycosylation step of protein maturation^16^, PrP^C^ progressively resolved into a single immunoreactive band of low molecular weight (Fig. 2b), corresponding to the non-glycosylated form (Supplementary Fig. 3A). Densitometry analysis revealed a slight but significant increase in PrP^C^ content in cells incubated with Tun for 24 h compared with vehicle-treated cells. As for Tun, the non-glycosylated form of PrP^C^ was overexpressed in cells undergoing glucose deprivation. However, total (Glycosylated plus non-glycosylated) PrP^C^ levels were reduced in glucose-deprived cells (Fig. 2c and Supplementary Fig. 3B). As expected, DTT, which affects disulfide bonds formation, did not affect PrP^C^ glycosylation profile (Fig. 2d and Supplementary Fig. 3C), whereas BFA produced a partially glycosylated protein, consistent with a blockade of transit through the Golgi network (Fig. 2e and Supplementary Fig. 3D). Thus, the pattern of PrP^C^ protein expression and maturation depends on whether the ER stress inducer affects protein maturation or ER function (Fig. 2f). Of note, western blot analysis of PrP^C^ in extracts from primary HREC exposed to Tg, Tun or BFA (Supplementary Fig. 1B) confirmed the quantitative and qualitative changes monitored with immortalized HREC.

**Fig. 3:**
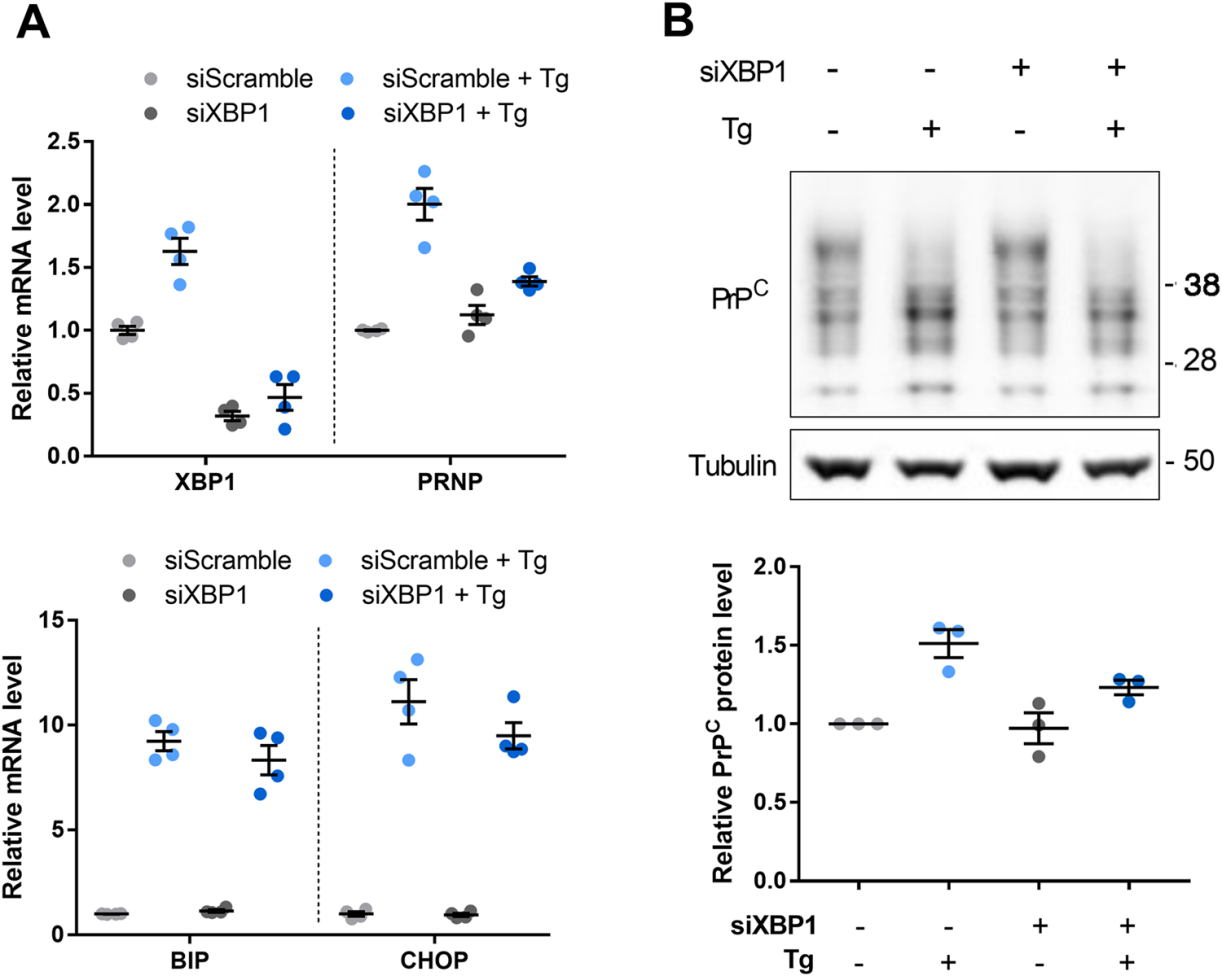
ER stress-induced *PRNP* expression is dependent on XBP1. **a** qRT-PCR analysis of the expression of *XBP1* and *PRNP* (top panel) as well as *BIP* and *CHOP* (bottom panel) in *XBP1*-silenced vs. control HREC exposed or not to Tg for 24 h. **b** Western blot analysis of PrP^C^ protein expression in *XBP1*-silenced vs. control HREC exposed or not to Tg for 24 h. Protein levels were normalized to Tubulin. Results are represented as individual values with means of *n* = 2 independent duplicates of cell preparations or *n* = 3 independent experiments ± SEM. Mean and 95% IC relative PrP^C^ expression levels: 200% [160–240] in the Tg^+^siXBP1^−^ condition, and 139% [127–150] in the Tg^+^siXBP1^+^ condition.

Since the *PRNP* promoter contains ER stress response elements^17,18^, we next assessed a potential contribution of XBP1 to the upregulation of *PRNP* mRNA in response to Tg. XBP1 silencing in HREC did not induce any change in the basal levels of *PRNP* mRNA and PrP^C^ protein, in the absence of ER stressor. In contrast, the silencing of XBP1 drastically reduced the upregulation of *PRNP* mRNA and PrP^C^ protein triggered by Tg (Fig. 3a top panel and B), while it did not affect the induction of *BIP* and *CHOP* expression, which depend on the activity of the ATF6 and PERK pathways of the UPR respectively (Fig. 3a bottom panel). Together, these results indicate that *PRNP* mRNA is induced in response to a variety of ER stressors in HREC and that this induction is XBP1-dependent at least in the case of Tg.

### ER stress promotes PrP^C^ secretion by HREC

**Fig. 4:**
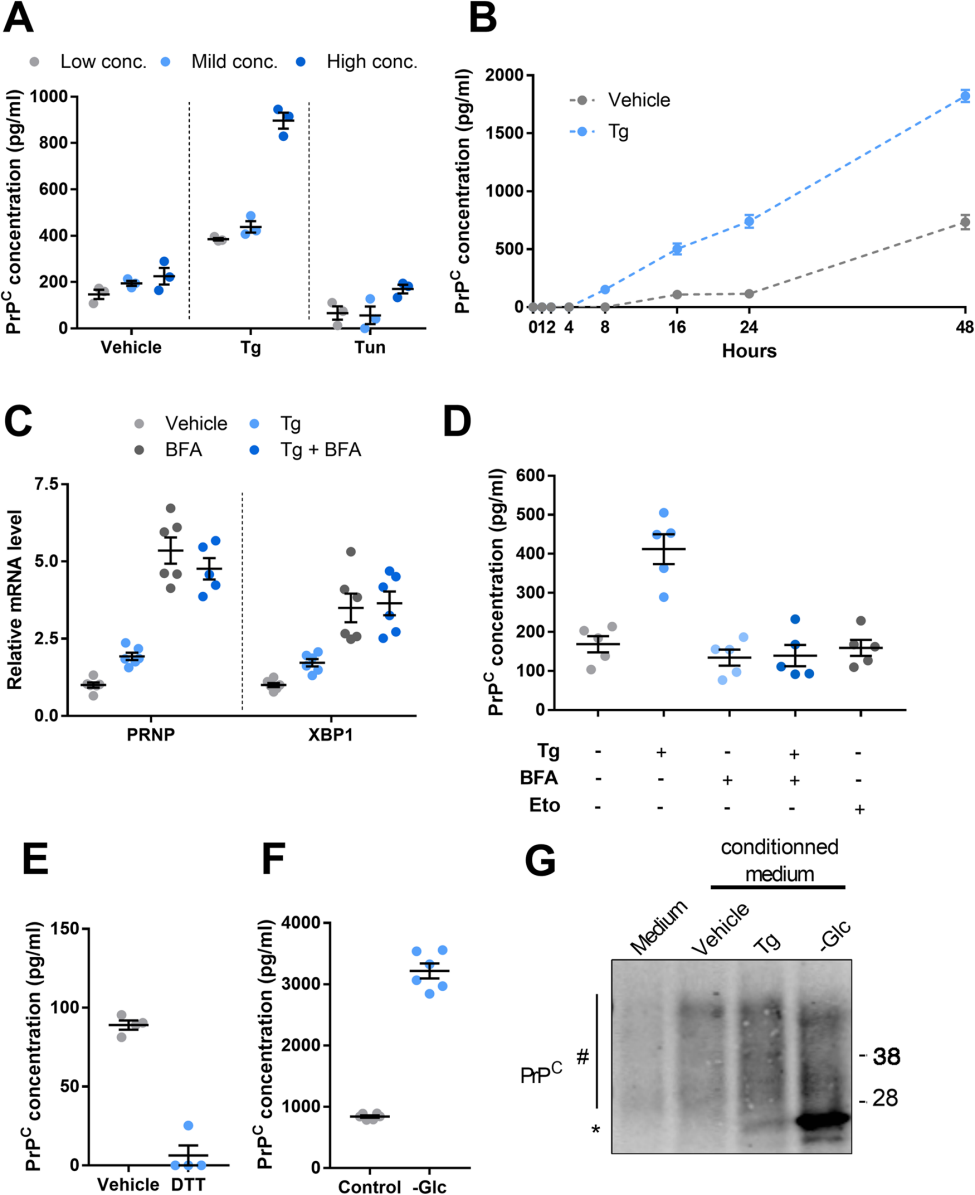
Impact of ER stressors on soluble PrP^C^. **a**, **b** Soluble PrP^C^ was measured in the conditioned medium of HREC exposed to increasing doses of Tg (Low:100 nM, Mild: 250 nM and High: 2.5 µM) or Tun (Low:1.25 µg/ml, Mild: 2.5 µg/ml and High: 5 µg/ml) for 24 h (**a**) or treated with 250 nM Tg during a 48-hour kinetics (**b**). **c** qRT-PCR analysis of *PRNP* mRNA expression in HREC exposed to 250 nM Tg in combination with 5 µg/ml BFA for 24 h. **d**, **e** Measurement of soluble PrP^C^ in the conditioned medium of HREC exposed to 250 nM Tg in combination with 5 µg/ml BFA or to 100 µM Etoposide for 24 h (**d**), in the conditioned medium of HREC exposed 1 µM DTT for 24 h (**e**) or submitted to glucose deprivation for 48 h (**f**). **g** Comparative migration profile of PrP^C^ protein immunoprecipitated from 200 µl of fresh culture medium or conditioned media from HREC treated with Tg for 24 h or vehicle or submitted to glucose deprivation for 48 h. *Hash* indicates mono- or bi-glycosylated PrP^C^ isoforms. *Asterisk* indicates unglycosylated PrP^C^. Results are represented as individual values with means of *n* = 2 independent duplicates or triplicates of cell preparations or *n* = 3 independent experiments ± SEM.

Since PrP^C^ can be found as a soluble form in addition to its major location at the cell surface^9^, we examined whether it could be secreted by renal epithelial cells in response to ER stress. To this aim, HREC were exposed for 24 h to various concentrations of Tg, which induce dose-dependent increases in UPR-associated transcripts (Supplementary Fig. 4A) and soluble PrP^C^ was quantified by ELISA in the extracellular medium. We observed a dose-dependent increase in soluble PrP^C^ in the supernatant of cells exposed to Tg, reaching almost 400% of control for the maximal dose (Fig. 4a). While the epitope recognized by the ELISA PrP^C^ quantification test is not disclosed, our preliminary data (not shown) suggest that soluble PrP^C^ species in the supernatant of HREC mainly correspond to N-terminally truncated isoforms^19^. To shed some light on the kinetics of soluble PrP^C^ accumulation, we collected supernatants from Tg- or vehicle-incubated HREC over a 48 h-time-frame. Soluble PrP^C^ concentration increased between 24 hours and 48 h in vehicle-exposed cells, supporting the occurrence of a basal shedding of PrP^C^ in the absence of stress (Fig. 4b). Under exposure to Tg at 250 nM, the levels of soluble PrP^C^ steadily accumulated from 8 h of drug addition onwards, reaching about 1800 pg/ml corresponding to 250% of control at 48 h (Fig. 4b).

As a GPI-anchored molecule, PrP^C^ carries a signal sequence for translocation in the ER lumen and follows the conventional secretory pathway^20^. The hallmark of conventional protein secretion is the trafficking through the ER-Golgi network, a process inhibited by Brefeldin A (BFA)^21^. Because of the disruption of the secretory pathway, BFA is also an ER stress inducer. In agreement, as observed with Tg and other ER stressors, BFA induced the expression of *PRNP* mRNA (Figs. 1c and 4c) and UPR-associated transcripts among which XBP1 (Fig. 4c and Supplementary Fig. 4B). However, when we monitored PrP^C^ secretion by HREC incubated with BFA, PrP^C^ could not be detected in the extracellular medium, suggesting that its secretion is inhibited when ER stress is generated by BFA (Fig. 4d). Importantly, Tg-induced PrP^C^ secretion was inhibited by BFA despite the fact that PrP^C^ transcripts were still expressed under Tg exposure (Fig. 4c, d). These results indicate that the intracellular trafficking of PrP^C^ during ER stress is altered by BFA, and that PrP^C^ is likely secreted through the canonical path under ER stress. In line with this, Etoposide, which promotes DNA damage and cell death without ER stress in our experimental system^22^, did not induce PrP^C^ secretion, indicating that PrP^C^ release is not a non-specific consequence of cell death and membrane permeabilization, and corroborates our finding supporting that PrP^C^ secretion is selectively controlled by ER stress (Fig. 4d).

When cells were exposed to Tun (Fig. 4a) or DTT (Fig. 4e) instead of Tg, there was no increase in soluble PrP^C^ whatever the dose, despite the induction of *PRNP* and UPR-associated transcripts in ER-stressed cells (Supplementary Fig. 4C, D). This is in line with the observed impact of Tun (Fig. 2b) on PrP^C^ maturation and may be accounted for by the necessity of PrP^C^ to undergo proper glycosylation to reach the cell surface. In addition, the reported effect of DTT on PrP^C^ maturation^23^ and the lack of impact of DTT on PrP^C^ glycosylation (Fig. 2f) indicate that PrP^C^ must undergo disulfide bond formation to exit the ER toward the Golgi apparatus, and that PrP^C^ lacking disulfide bond is likely directed towards the proteasome through the ER associated degradation pathway^24^. However, 48 hours of glucose deprivation, which induces ER stress and prevents PrP^C^ glycosylation in HREC (Supplementary Fig. 3B, F) was associated with a prominent increase (3.8-fold, 3200 pg/ml) in soluble PrP^C^ level compared to the control condition (Fig. 4f). The apparent discrepancy between the effects of Tun and glucose deprivation on PrP^c^ secretion may be partly due to quantitatively different effects on PrP^c^ glycosylation. Tun blocks N-linked glycosylation by inhibiting N-acetylglucosamine phosphotransferase, which catalyzes the first step of the glycosylation process^25^. On the other hand, glucose starvation also affects glycosylation by reducing the pool of substrates available for *N*-acetylglucosamine-1-phosphate production^26^, but gluconeogenesis (from glucogenic aminoacids for example) might mitigate the global effects of glucose deprivation, thus enabling residual N-linked glycosylation activity. However, immunoblotting of culture-medium immunoprecipitated PrP^C^ suggested that a proportion of secreted PrP^C^ upon glucose deprivation was non/underglycosylated, suggesting that under this condition, non/underglycosylated proteins can escape ER quality controls and still be secreted (Fig. 4g).

### PrP^C^ silencing does not affect cell viability in ER-stressed HREC

**Fig. 5:**
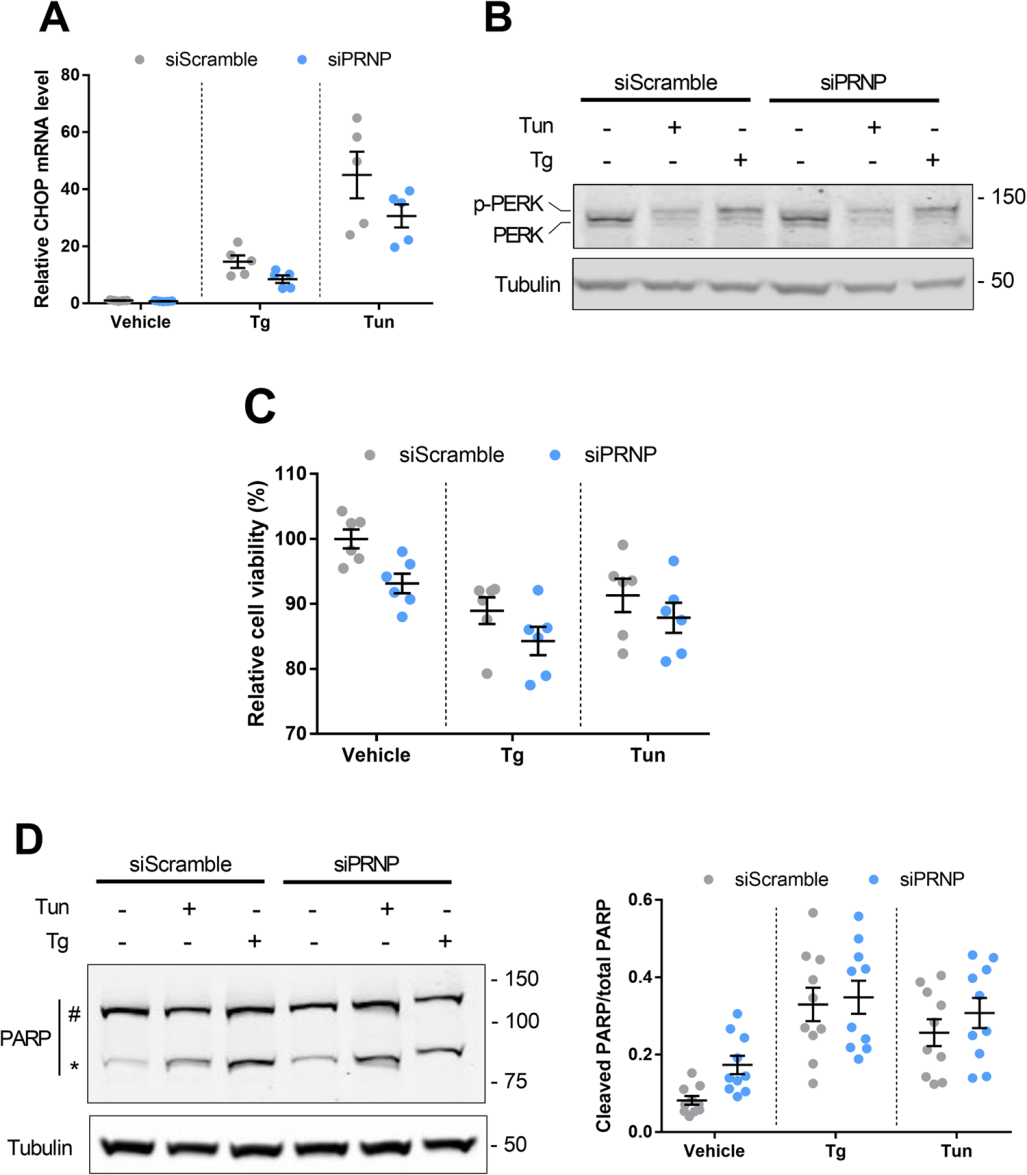
Impact of *PRNP* silencing on the response of HREC to ER stress. **a** qRT-PCR analysis of the expression of *CHOP* in *PRNP*-silenced vs. control HREC exposed or not to Tg or Tun for 24 h. Mean and 95% IC of relative CHOP mRNA levels: Tg^+^ siScramble: 4500% [2241–6760]; and Tg+ siPRNP: 3064% [1946–4183]. Tn^+^ siScramble: 1463% [849–2075] and Tn+ siPRNP: 851% [482–1219]. **b** Western blot analysis of the expression of PERK in *PRNP*-silenced vs. control HREC exposed or not to Tg or Tun for 24 h. **c** Relative cell viability of *PRNP*-silenced vs. control HREC exposed or not to Tg or Tun for 24 h. Mean and 95% IC of % cell viability: vehicle siScramble: 100% [96–103] and vehicle siPRNP: 89% [93–97]: Tg+ siScramble: 88%[93–94], and Tg+ siPRNP: 84 [78–89]; Tn^+^ siScramble: 91% [84–97] and Tn+ siPRNP: 87% [81–93]. **d** Western blot analysis of the expression of PARP (left) and quantification of the ratio cleaved PARP to total PARP (right) in *PRNP*-silenced vs. control HREC exposed or not to Tg or Tun for 24 h. Protein levels were normalized to Tubulin. Results are expressed as individual values with means of *n* = 5 (**a**), *n* = 6 (B,C) or *n* = 10 (**d**) independent experiments ± SEM.

We next sought to determine whether the induction of PrP^C^ upon ER stress affects cell viability. To this purpose, PrP^C^ expression was silenced in HREC by siRNA-mediated RNA interference for 24 h and cells were further exposed to Tg or Tun in the presence of siPRNP for another 24 h. PrP^C^-silenced HREC, which expressed only 20% *PRNP* mRNA and <35 % PrP^C^ protein (Supplementary Fig. 5A, B), retained the ability to mount a robust ER-stress response (Supplementary Fig. 5C). We nevertheless found that PrP^C^ silencing mitigated the Tun- or Tg-induced increase in CHOP mRNA levels in HREC (Fig. 5a). However, PERK phosphorylation, reflecting activation of the Integrated Stress Response that mediate CHOP expression was not affected by PrP^C^ silencing, suggesting that PrP^C^ could affect CHOP expression downstream PERK (Fig. 5b). Since CHOP is associated with the pro-apoptotic branch of the UPR^27^, this would suggest that PrP^C^ favors a pro-apoptotic fate in ER-stressed cells. This prompted us to compare the impact of ER stress on cell viability in PrP^C^-silenced HREC versus control HREC using a CASY.TT cell counter. In basal conditions, i.e. in the absence of Tun or Tg, we observed a significant reduction in cell viability upon PrP^C^ depletion (Fig. 5c), in line with the physiological pro-survival role of PrP^C^^9^. As expected, ER stressors reduced cell viability, but the extent of cell viability reduction in PrP^C^-silenced HREC incubated with Tun or Tg was not different from PrP^C^-expressing HREC (Fig. 5c). These results were corroborated by the cleavage of PARP, a proxy of apoptotic cell death^28^. Indeed, in non-treated cells, PrP^C^ silencing promoted an increase in cleaved PARP to total PARP, and ER stressors induced PARP cleavage that was comparable whether cells expressed PrP^C^ or not (Fig. 5d). Altogether, these results lend support to a pro-survival effect of PrP^C^ under basal conditions that would not necessarily be maintained upon cell stress, which is reminiscent of the ambivalent role described for this protein^29^.

### Urinary PrP^C^ as a potent non-invasive biomarker of AKI

**Fig. 6:**
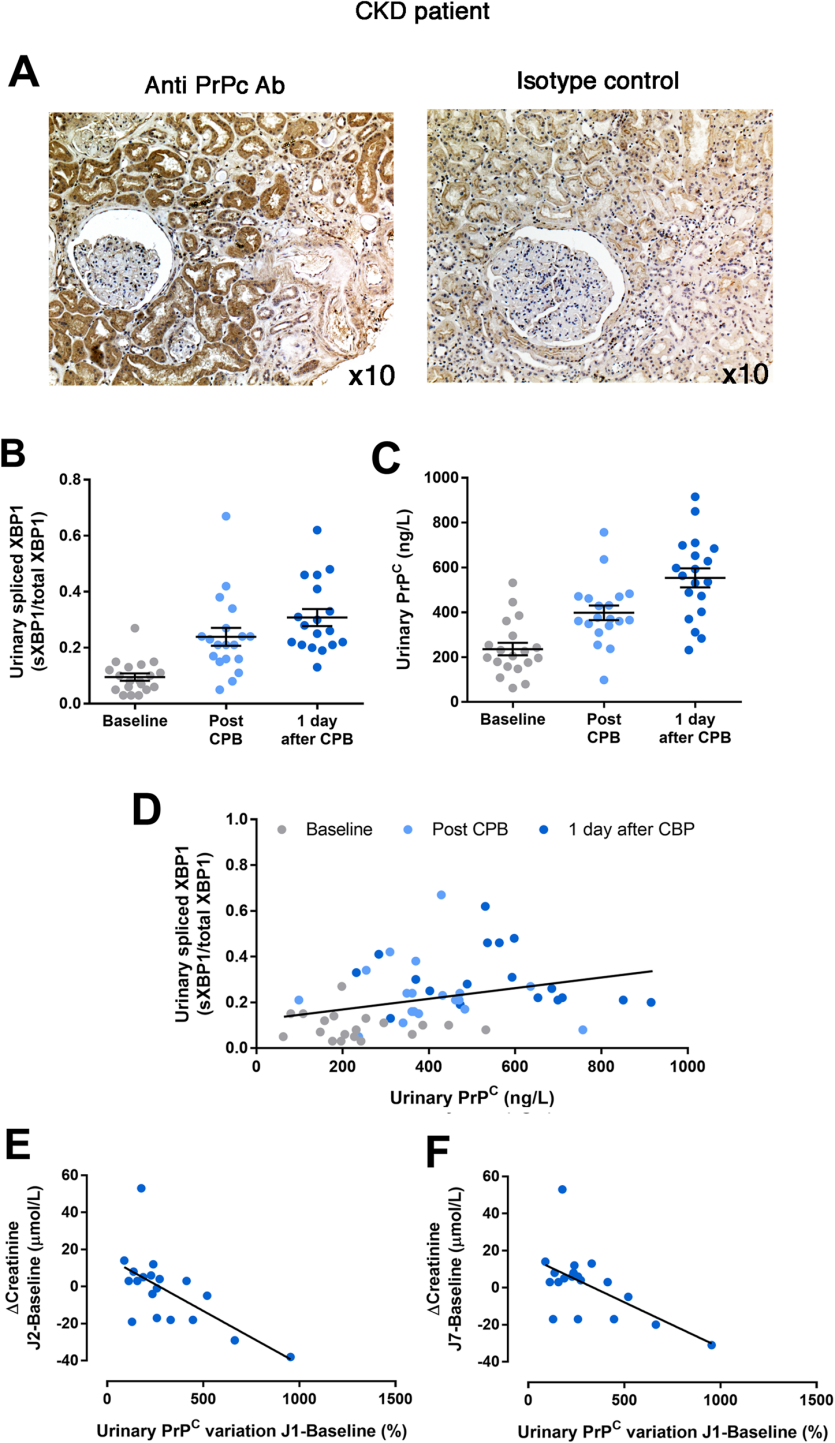
PrP^C^ is expressed in kidney and is released in urines of patients with hemodynamic impairment. **a** Representative photomicrograph of PrP^C^ expression evaluated by immunohistochemistry in a kidney from individual with chronic kidney disease (left panel). Staining with the isotype control antibody is shown in the right panel. Original magnification, ×10. **b**–**f** Measurement of relative *XBP1* splicing (**b**) and soluble PrP^C^ (**c**) in the urines of *n* = 19 patients undergoing CBP before, at the end and the day after surgery. **c** Linear regression curve between urinary PrP^C^ concentrations and sXBP1 mRNA levels in urines before, after and the day after CBP. **d**, **e** Correlation between PrP^C^ increase rate between the day 1 after CBP and baseline, and the variation of serum creatinine at day 2 (**d**) and day 7 (**e**) compared to baseline (before CBP).

Since PrP^C^ is secreted by the renal epithelium under stressful conditions in vitro, we wondered if it could be detected and quantified in urines of individuals with a renal tubular epithelial injury. Supporting this possibility, we found that PrP^C^ expression was expressed only in epithelial cells, and accumulated beneath the apical membrane of the cells, a feature suggestive of secretion process (Fig. 6a**)**. We then measured the concentrations of PrP^C^ in urines of individuals with a kidney disease. To demonstrate the biological and clinical relevance of the detection of PrP^C^ in urine, we monitored ER stress markers in urine of 19 patients undergoing scheduled cardiac surgery with cardiopulmonary bypass, which promote IRI in the kidney, a condition associated with ER stress (Supplementary Table 2)^12^. We repeatedly measured the urine angiogenin concentrations (an indirect marker of the activity of the IRE1α/sXBP1 axis^22^), and NGAL, which is regulated by the PERK/ATF4 pathway^30^, to obtain information about the activation of the UPR during the CPB procedure. In addition, we measured CHOP and sXBP1 mRNA in urine cell pellets. Urine angiogenin concentrations and the ratio of sXBP1/(sXBP1 + XBP1) increased significantly over time (before, immediately after and one day after CPB), confirming that hemodynamic impairment initiates ER stress and sXBP1 expression (Supplementary Fig. 6A and Fig. 5b). NGAL concentrations, and CHOP or BIP transcripts (Supplementary Fig. 6B, C and D) did not vary, indicating that the IRE1α/sXBP1 axis, rather than the PERK/ATF4 axis of the UPR, appears to be activated in the kidneys under these specific hemodynamic conditions. Finally, we observed an incremental increase in the concentrations of PrP^C^ after CPB, and a positive correlation between urinary sXBP1 transcripts levels and PrPc concentrations in urines (Fig. 6b, c), in line with the activation of the IRE1-sXBP1 pathway, which regulate PrP^C^ expression upon ER stress. Moreover, patients with the highest amplitude of PrP^C^ urinary concentrations between baseline and the day after CBP were those with the greatest recovery in serum creatinine, suggesting that the sXBP1-PrP^C^ pathway is likely cyto and nephroprotective (Fig. 6d). Together, these results indicate that PrP^C^ is a potent non-invasive marker of kidney injury reflecting ongoing ER stress in the kidney.

### Urinary PrP^C^ as a potent non-invasive biomarker of CKD

**Fig. 7:**
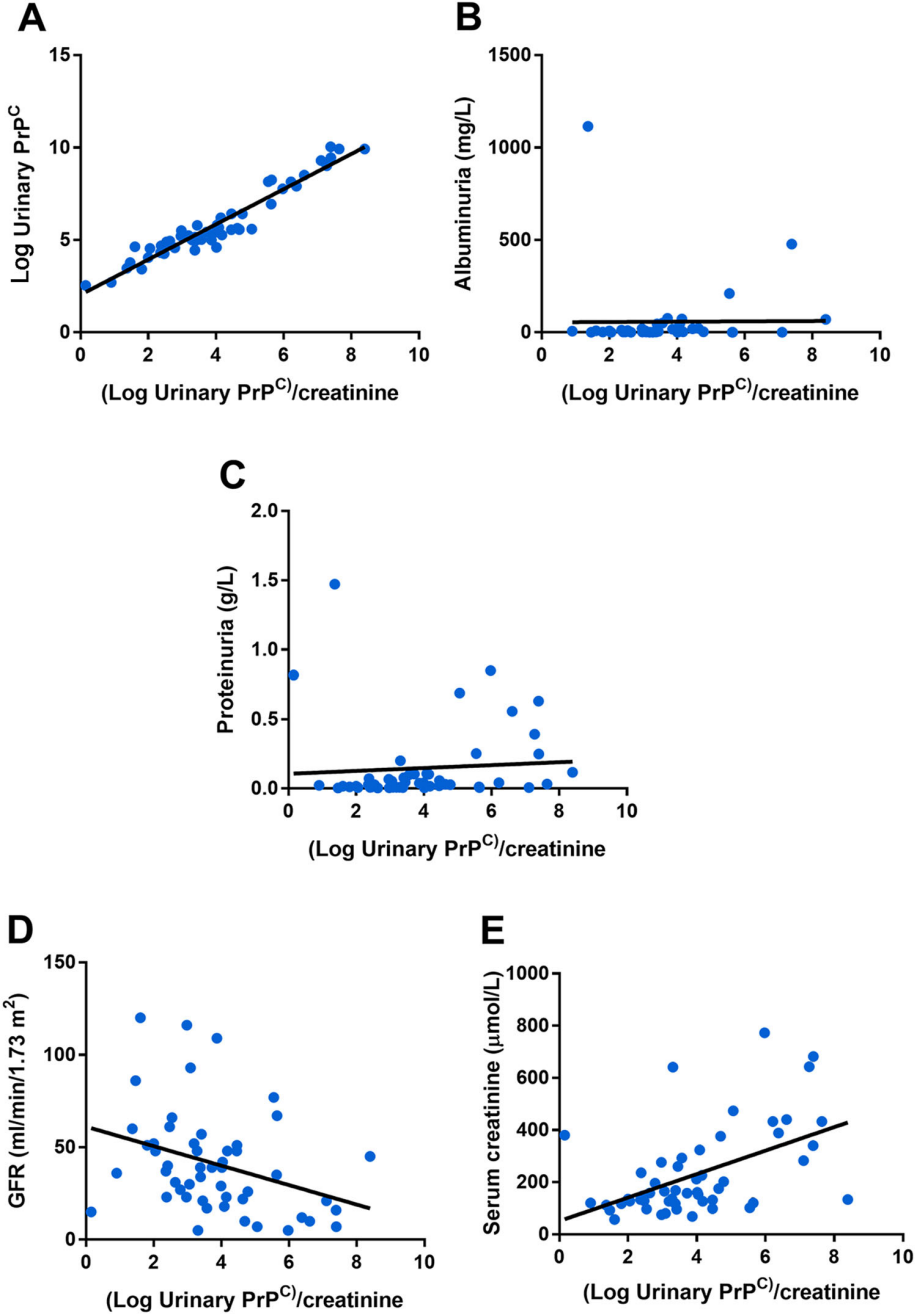
Urinary PrP^C^ is elevated in patients with CKD. **a**–**f** Log-transformed values of urinary PrP^C^ reported to urinary creatinine concentrations and plotted as a function of urinary PrP^C^ (*R*^2^ = 0.92) (**a**) albuminuria (*R*^2^ = 0) (**b**), proteinuria (*R*^2^ = 0) (**c**), glomerular filtration rate (*R*^2^ = 0.11) (**d**) or serum creatinine (*R*^2^ = 0.24) (**e**) in a cohort of *n* = 55 patients with CKD.

We next aimed at providing insights into the specificity of urinary PrP^C^ in regards with proteinuria and renal function. To this end, we measured the concentrations of PrP^C^ in the urines of 55 consecutive patients referred to a nephrology department for a CKD (defined by presence of significant proteinuria and/or an estimated Glomerular filtration rate < 60 mL/min/1.73 m^2^) (Supplementary Table 3). PrP^C^ concentration was reported to the urinary concentrations of creatinine to avoid bias related to variations in urine concentration (Fig. 7a). Because the distribution of PrP^C^ was skewed, we log-transformed the values to obtain a Gaussian distribution and compared PrP^C^ concentration with other parametric variables. We tested the correlation between the levels of urinary PrP^C^, the high molecular weight protein albumin (a marker of glomerular injury), and total proteinuria. Indeed, the presence of PrP^C^ in urines could be a non-specific consequence of increased glomerular permeability, and therefore a component of total proteinuria. We found that the urinary concentrations of PrP^C^ did not correlate with total urinary proteins and albuminuria (Fig. 7b, c). However, urinary PrP^C^ concentrations were positively correlated with serum creatinine (*R*^2,^ 0.25, *p* < 0.0001) and inversely correlated with estimated glomerular filtration rates (*R*^2,^ 0.11, *p* < 0.01), two markers of renal function (Fig. 7d, e), suggesting that in patients with CKD, chronically injured tubules secrete PrP^C^ and that PrP^C^ concentrations could reflect the severity or the extent of the injury. These results indicate that the detection and quantification of PrP^C^ in urine could provide information on renal function.

## Discussion

This proof-of-concept study is the first to demonstrate that PrP^C^ is a potent non-invasive, and mechanistic biomarker for ER stress-mediated kidney diseases. Aberrant ER protein proteostasis and ER dysfunction underpin the initiation and development of a variety of kidney diseases, and it is of outmost importance to identify and validate ER stress biomarkers that can be applied in human kidney disease patients. Here, we demonstrate that PrP^C^ is rapidly upregulated and secreted when renal cells were subjected to ER stress. Moreover, PrP^C^ is detectable and easily quantifiable in urine of patients with CKD, and correlates with renal function. Finally, we validated the potent clinical utility of PrP^C^ as a marker of ER stress in patients with kidney IRI.

ER-stressed cells produce signals that alert neighboring cells of the presence of a stressor, and an emerging concept is that the secretome produced by stressed cells, including the mediators secreted in urines by the renal epithelial cells upon kidney injury, can constitute the fingerprint ER stress. Hence, a deep molecular characterization of this secretome appears as a valuable approach for the identification of soluble markers of an ongoing tissue injury associated with ER stress. The use of biomarkers of ER stress in the kidney has promising applications. Since ER stress is associated with the underlying pathogenesis of kidney diseases, mechanistic ER stress markers could allow the initiation of treatments early on in the disease, which will reduce the follow-up time to identify kidney disease with serum creatinine alone. Indeed, serum creatinine elevations occur when irreversible changes such as scarring and fibrosis of the kidney are often present, and treatment is unlikely to restore full kidney function.

Critically, monitoring urinary PrP^C^ allows an early detection of tubular ER stress, an underlying causative mechanism for ischemic AKI. Approximately 45% of critically ill patients and 20% of hospitalized patients develop AKI, which leads to increased hospital stays, and increased morbidity and mortality^31^. Our translational studies in patients undergoing CPB surgery suggest that PrP^C^ could be utilized to stratify the risk of developing AKI after IRI, which remains to be demonstrated further investigations. The ultimate utility of PrP^C^ may be to identify an early therapeutic window during which tubular ER stress modulators can be applied before a detectable change in renal function occurs. We took advantage of the CBP cohort to assess potential correlations between changes in urinary PrP^C^ levels and clinical correlates in patients. PrP^C^ levels correlate with those of spliced XBP1, in line with our in vitro results demonstrating that the increase in *PRNP* gene expression in response to ER stress depends on XBP1. Furthermore, we found that the variation of PrP^C^ levels within the first 24 h of CPB is inversely correlated to the variation of creatinine levels within the first 48 h of CPB. This also holds true when considering the variation levels of creatinine within the first week of CPB. This hence suggests that patients who mount a robust PrP^C^ response (high fold change in PrP^C^ levels versus baseline) are those whose kidney function recovers better/faster (highest rate of creatinine reduction). This would fit in with the protective role ascribed to PrP^C^ in the context of various types of stresses (see ref. ^9^ for review). Conversely, in the context of CKD, our results suggest that high PrP^C^ levels are associated with worse renal function. The reasons for the differences between acute and chronic injuries are probably multiple and remain to be established. However, one can speculate that the production of PrP^C^ during CKD reflects chronic ER stress, which is known to be deleterious since it is associated with the engagement of proapoptotic pathways. In addition, deleterious stress pathways that are unrelated to ER stress could be engaged upon CKD, leading to PrP^C^ production. Finally, PrP^C^ by itself could promote tubular cell death and activate fibrogenesis, that would ultimately drive chronic structural deterioration and CKD progression.

In addition to AKI, autosomal dominant tubular kidney disease (ADTKD) are monogenic conditions in which ER stress is prominent and leads to tubulointerstitial fibrosis and progression of CKD. ADTKD are phenotypically heterogeneous with variable age of disease onset, disease severity, and rate of disease progression among affected individuals within and between families. Remarkably, mutations in the *UMOD* gene encoding Uromodulin, which is the most abundant protein secreted in normal urine and has multiple roles in kidney physiology, as well as mutations in the *MUC1* gene which encodes the transmembrane epithelial mucin 1 protein supporting roles in epithelial barrier protection, lead to mutant proteins retention and aggregation in the ER, likely due to protein misfolding. The activation of the UPR in those inherited diseases leads to progressive tubular damage, triggering inflammation and interstitial fibrosis^32,33^. The identification and validation of ER stress markers is extremely important in this area, as it will provide a useful tool for diagnostic purpose and monitoring disease activity.

A set of molecules released or secreted by renal cells under ER stress in urine has been recently described, and the proof-of-concept of a non-invasive monitoring of renal ER stress in human has been done for some of them. For example, ERp57 (also known as PDIA3), is secreted by ER stressed cells involved in TGF-β mediated fibrogenesis, and can be detected in urines of patients with diabetic nephropathy with ongoing fibrosis^34^. Another example is the ER chaperone mesencephalic astrocyte-derived neurotrophic factor (MANF), which is secreted by ER stressed tubular cells and can be quantified in the urine of tunicamycin-treated mice^35^. The secreted ribonuclease angiogenin is expressed under the control of IRE1α, thereby reflecting the activation of the UPR in the kidney. Urinary angiogenin levels reflect the severity of tissue damage upon acute kidney allograft injury and are predictive of graft failure, independent of histological lesions^36^. Supporting a more generalized role of the UPR transducers in renal disease, the PERK pathway, through the expression of lipocalin 2, regulates tubular cell viability upon proteinuric stress, and lipocalin 2 when secreted in urines can provide prognostic information^30,37^. Reflecting the activity of the ATF6 arm of the UPR, cysteine-rich with EGF-like domains 2 (CRELD2) is upregulated and secreted when renal cells are exposed to ER stressors and appears to be a potential urine ER stress biomarker in patients with kidney diseases associated with ER stress including ADTKD-UMOD and ischemic AKI^38^.

While considerable attention has been paid to PrP^C^ for its involvement in neurodegeneration, very little is known on its contribution to kidney pathophysiology. PrP^C^ is expressed at the apical cell surface of polarized proximal tubule cells and promotes kidney iron uptake^39^. Interestingly, PrP^C^ is upregulated in the kidney following IRI^40^, in line with our own data in CBP patients, and the results indicate that PrP^C^-knockout animals have an increase sensibility to IRI. However, the authors did not assess the link with the UPR nor the levels of urinary PrP^C^ following IRI. In contrast, tissue damage was increased in PrP^C^-knockout animals under IRI, which could be accounted for by a reduction in *Xbp1* transcripts in PrP^C^-knockout vs. WT animals (our unpublished data). While the induction of PrP^C^ in ER stressed cells has been described^18^, the impact of ER stress on soluble PrP^C^ had not been investigated. Besides, studies were mainly carried out on neurons or cancer cells and have highlighted a protective role for PrP^C^ against ER stress-induced cell death. Thus, the functional outcome of ER stress-induced PrP^C^ upregulation may depend on the cell type, tissue considered and duration (acute versus chronic). We believe the connection between PrP^C^ and ER stress may be of particular relevance in the context of kidney in view of our data obtained in patients with acute or chronic kidney injury. Furthermore, our unpublished data indicate a strong correlation between *PRNP* gene expression and the UPR as well as XBP1-dependent transcription in kidney renal clear cell carcinoma. Incidentally, PrP^C^ overexpression has been reported in several types of cancer^41^ and a consensus view is that increased levels of PrP^C^ endow cells with proliferative^42,43^ migratory and invasive capacities^44–46^, which are also features supporting tissue remodeling in CKD^3,47^. ER stressed cells shape tissue remodeling since cells upon ER stress secrete numerous specific mediators that promote inflammation, such as Interleukins 6 and 8 and Chemokine (C-X-C) Ligand 3 (CXCL3); angiogenesis through Vascular Endothelial Growth Factor (VEGF) and basic Fibroblast Growth Factor (bFGF); tissue remodeling through the production of collagen 1A2 and the metalloprotease ADAMTS3; apoptosis through the production of Prostate Apoptosis Response 4 (Par-4); and proteostasis with the chaperon ERdj3, which is a critical regulator of tissue homeostasis^1^. Whether PrP^C^ participate in tissue remodeling upon acute and chronic kidney diseases is unknown and is an important research avenue.

In summary, our study has identified PrP^C^ as a mechanistic urine biomarker in ER stress-mediated kidney diseases. The clinical applications of PrP^C^ in ER stress-mediated kidney diseases may include early diagnosis, stratification of patients at risk and monitoring treatment response. Further studies in larger cohorts are required to evaluate the diagnostic accuracy and predictive value of PrP^C^.

## Supplementary information

Supplementary figures legends

Supplementary tables

S1

S2

S3

S4

S5

R6
