# Supplementary material for: The cellular prion protein is a stress protein secreted by renal tubular cells and a urinary marker of kidney injury
Source: Cell Death Dis. 2020 Apr 17;11(4):243. doi: 10.1038/s41419-020-2430-3 (PMC7165184; doi:10.1038/s41419-020-2430-3)
Supplement: Supplementary file 1 — Supplementary figures legends [file 41419_2020_2430_MOESM1_ESM.docx]

**Supplementary information**

**Supplementary figure legends**

**Fig. S1: Impact of ER stressors on *PRNP* mRNA and PrP^C^ protein expression in primary HREC**. (A) qRT-PCR analysis of *BIP*, *CHOP*, *GADD34*, *XBP1*, (left panel) or *PRNP* (right panel) mRNA expression in primary HREC incubated with 250 nM Tg, 2.5 µg/ml Tun or 5µg/ml BFA for 24h. (B) Western blot analysis (left panels) and quantification (right panels) of PrP^C^ or BIP proteins in primary HREC incubated with 250 nM Tg (top panel), 2.5 µg/ml Tun (middle panel) or 5 µg/ml BFA (bottom panel) for 24h. Tubulin is used as a loading control. Results are represented as individual values with means of n=2 independent duplicates of cell preparations ± SEM.

**Fig. S2: Impact of various ER stressors on PrP^C^ protein expression and glycosylation**

**A.** Graph representing the relative expression (means±sem) of BiP, CHOP, GADD34, sXBP1 and PRNP transcripts measured through RT-qPCR in kidneys of 3 mice treated with 1 mg/kg tunicamycin for 48 hours.

**Fig. S3: Impact of various ER stressors on PrP^C^ protein expression and glycosylation.** (A) Comparative migration profile of PrP^C^ in control cells or in cells exposed to Tun for 24h after deglycosylation of protein extracts with PNGaseF. # mono- or bi-glycosylated PrP^C^ isoforms. * unglycosylated PrP^C^. (B-D) Representative images of Western blot analysis of PrP^C^ and BIP protein expression in HREC submitted to glucose deprivation for 48h (B) or treated with DTT (C) or BFA (D) for 24h. Tubulin is used as a loading control.

**Fig. S4: Impact of various ER stressors on UPR genes.** (A) qRT-PCR analysis of *BIP*, *CHOP* and *GADD34* mRNA expression in HREC exposed to increasing doses of Tg. (B) qRT-PCR analysis of *BIP*, *CHOP* and *GADD34* mRNA expression in HREC exposed to 250 nM Tg in combination with 5 µg/ml BFA for 24h. (C) qRT-PCR analysis of *BIP*, *CHOP* and *GADD34* mRNA expression in HREC exposed to increasing doses of Tun. (D) qRT-PCR analysis of *BIP*, *CHOP* and *GADD34* mRNA expression in HREC exposed to 1 µM DTT for 24h. Results are expressed as individual values with means of at least 3 independent experiments ± SEM.

**Fig. S5: *PRNP* silencing in HREC and response to ER stress.** (A,B) qRT-PCR and western blot analysis of the expression of *PRNP* mRNA (A) and PrP^C^ protein (B) levels in *PRNP*-silenced vs. control HREC exposed or not to Tg or Tun for 24h. (C) qRT-PCR analysis of the expression of *BIP* (left) or *GADD34* (right) mRNA in *PRNP*-silenced vs. control HREC exposed or not to Tg or Tun for 24h. Results are expressed as means of n=2 independent duplicates or triplicates of cell preparations ± SEM..

**Fig S6.** **ER stress markers in urines of patients with hemodynamic impairment**. (A) Measurement of soluble Angiogenin, or (B) NGAL concentration, (C) *CHOP* and (D) *BIP* mRNA levels (D) in the urines of n=19 patients undergoing CBP before, at the end and the day after surgery.
