# Supplementary material for: The cellular prion protein is a stress protein secreted by renal tubular cells and a urinary marker of kidney injury
Source: Cell Death Dis. 2020 Apr 17;11(4):243. doi: 10.1038/s41419-020-2430-3 (PMC7165184; doi:10.1038/s41419-020-2430-3)
Supplement: Supplementary file 2 — Supplementary tables [file 41419_2020_2430_MOESM2_ESM.docx]

**Supplementary Table 1.**

| **List of human primers used** | |  |
| --- | --- | --- |
|  |  |  |
|  | Forward | Reverse |
| BIP | GGTGAAAGACCCCTGACAAA | GTCAGGCGATTCTGGTCATT |
| CHOP | TGGAAGCCTGGTATGAGGAC | TGTGACCTCTGCTGGTTCTG |
| GADD34 | AGGAGGCTGAAGACAGTGGA | GGCCATCTGCAAATTGACTT |
| PRNP | CGAGCTTCTCCTCTCCTCAC | GTTCCATCCTCCAGGCTTC |
| RPL13A | CCTGGAGGAGAAGAGGAAAGAGA | GAGGACCTCTGTGTATTTGTCAA |
| XBP1 | GCAGGTGCAGGCCCAGTTGT | CACACAGGATGGCTTGAAG |
|  |  |  |
|  |  |  |
| **List of rodent primers used** | |  |
| Bip | GGAGGAGGACAAGAAGG | ACTACGACGGTGTGATG |
| Chop | CGACAGAGCAAAATAACAGC | GGATGCAGGGTCAAGAGTAGTG |
| Gadd34 | GCTGGGTCCTTACCTTACCC | AGGGAGTGGTCACATCTTGG |
| Prnp | TTGGCAACGACTGGGAGGAC | GGACTCCTTCTGGTACTGGGTGA |
| Tbp | CAAACCCAGAATTGTTCTCCTT | ATGTGGTCTTCCTGAATCCCT |
| Xbp1 | CTGAGTCCGCAGCAGGTGCAG | CTCTGGGGAAGGACATTTCA |

**Supplementary Table 2.** Description of the cohort undergoing cardiac surgery

| **Characteristics (n=19)** |  |
| --- | --- |
| Age (years) | 62.5±18.1 |
| Men, n (%) | 12 (63) |
| BMI (kg/cm^2^) | 24.4±4.3 |
| Preexisting medical condition, n (%)   - Hypertension - Diabetes - Obesity - Atherosclerotic disease - CKD | 11 (57)  1 (5)  1 (5)  2 (10) |
| Preoperative characteristics   - eGFR (ml/min/1.73 m^2^) - Ejection fraction (%) - Cleveland score - Euroscore 2^$^ | 73.7±22.5  58±10  2.56±1.5  3±3.5 |
| Preoperative medications, n (%)   - RAS inhibitors - Diuretics - Beta blockers | 10 (52)  4 (21)  12 (63) |
| Indications, n (%)   - CABG - Mitral valve - Aortic valve - Ascending aorta | 3 (15)  3 (15)  10 (52)  3 (15) |
| Operative parameters   - CBP length (min) - Aortic cross-clamp length (min) - Whole procedure length (min) - Fluid loading (ml) - Blood loss (ml) - Cell Saver (ml) - Per CBP urine output (ml/kg/h) - Per procedure urine output (ml/kg/h) | 103.2±39.6  77±32.2  292.1±50.1  1715±712  2998±1977  971±870  120.6±139  258±133 |
| Post-operative parameters   - KDIGO AKI - Dialysis, n (%) - ICU stay (days) - Hospital stay (days) - Death, n (%) | 2 (10)  1 (5)  3.4±4.5  15.5±10  1 (5) |

Plus-minus values are means±standard deviation.

BMI denotes body mass index; CKD, chronic kidney disease; eGFR, estimated glomerular filtration rate; RAS, renin-angiotensin system; CABG, coronary artery bypass grafting; CPB, cardiopulmonary bypass; KDIGO, Kidney Diseases-Improving Global Outcome; ICU, intensive care unit.

**Supplementary Table 3.** Description of the cohort with chronic kidney disease

| **Characteristics (n=55)** |  |
| --- | --- |
| Age (years) | 64.1±34.1 |
| Men, n (%) | 39 (71) |
| BMI (kg/cm^2^) | 27.2±6.3 |
| Primary kidney disease   - Diabetes - Hypertension - Glomerulonephritis - MGRS - Hereditary - Nephrotoxicity - Other - Unknown origin | 9 (16.5)  7 (13)  11 (20)  4 (7)  5 (9)  3 (5.5)  9 (16)  7(12) |
| Kidney transplant recipient, n (%) | 19 (34) |
| Preexisting medical condition, n (%)   - Hypertension - Diabetes | 41 (75)  19 (35) |
| Nephrotoxic drugs, n (%) | 34 (62) |
| Baseline clinical chemistry   - Serum creatinine - eGFR (ml/min/1.73 m^2^) - eGFR loss/year (ml/min/1.73 m^2^) - PCR (g/g) - ACR (mg/g) - Hematuria (10^3^/ml) - Leucocyturia (10^3^/ml) | 229±168  40.7±28.4  0.22±9.6  2.7±2.8  56.6±181.1  10±40  6±8 |

Plus-minus values are means±standard deviation.

BMI denotes body mass index; MGRS, monoclonal gammopathy of renal significance; eGFR, estimated glomerular filtration rate; PCR, protein-to-creatinine ratio; ACR, albumin-to-creatinine ratio.
