# Supplementary figures and images for: The cellular prion protein is a stress protein secreted by renal tubular cells and a urinary marker of kidney injury
Source: Cell Death Dis. 2020 Apr 17;11(4):243. doi: 10.1038/s41419-020-2430-3 (PMC7165184; doi:10.1038/s41419-020-2430-3)

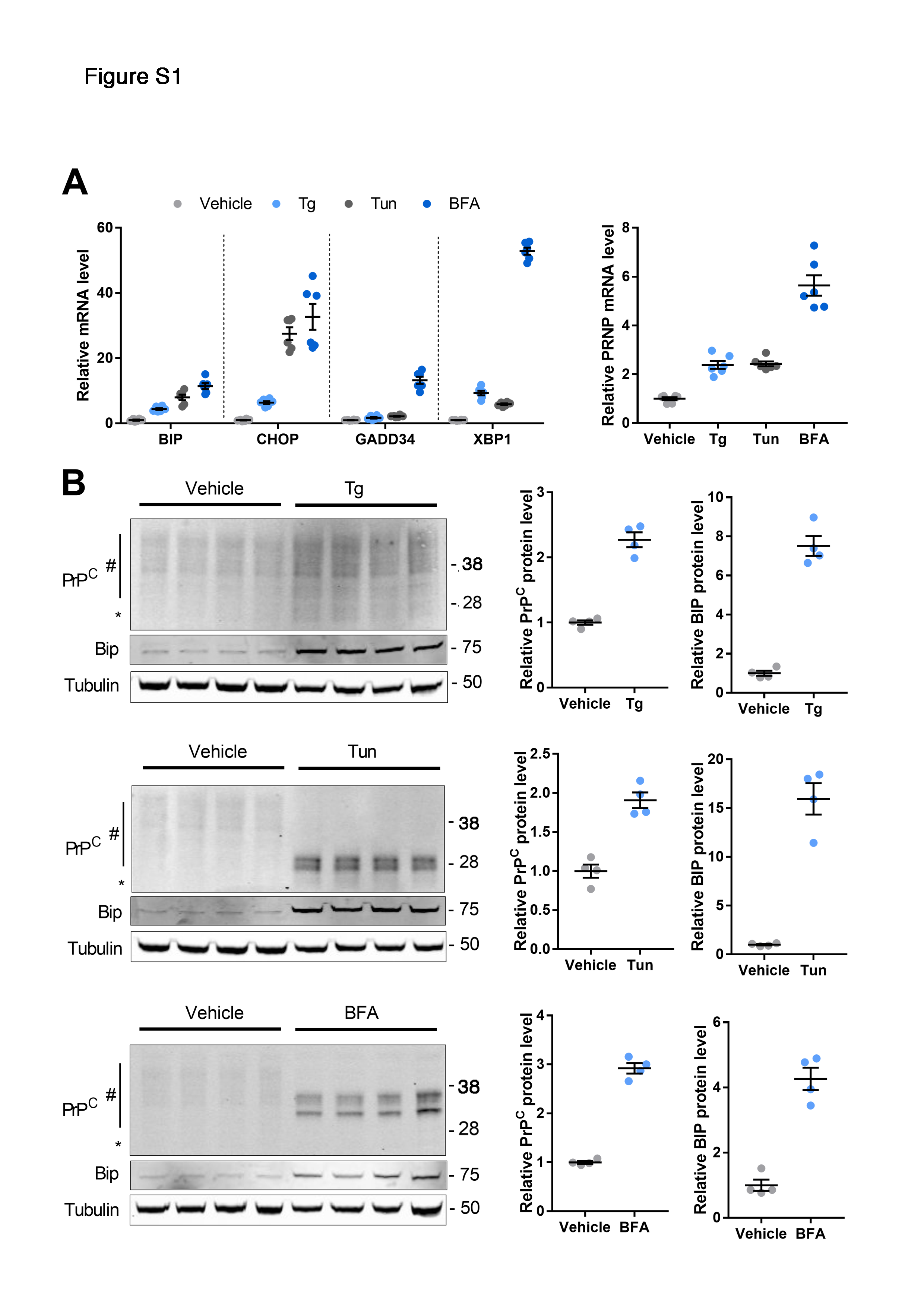

Supplement: Supplementary file 3 — S1 [file 41419_2020_2430_MOESM3_ESM.png]

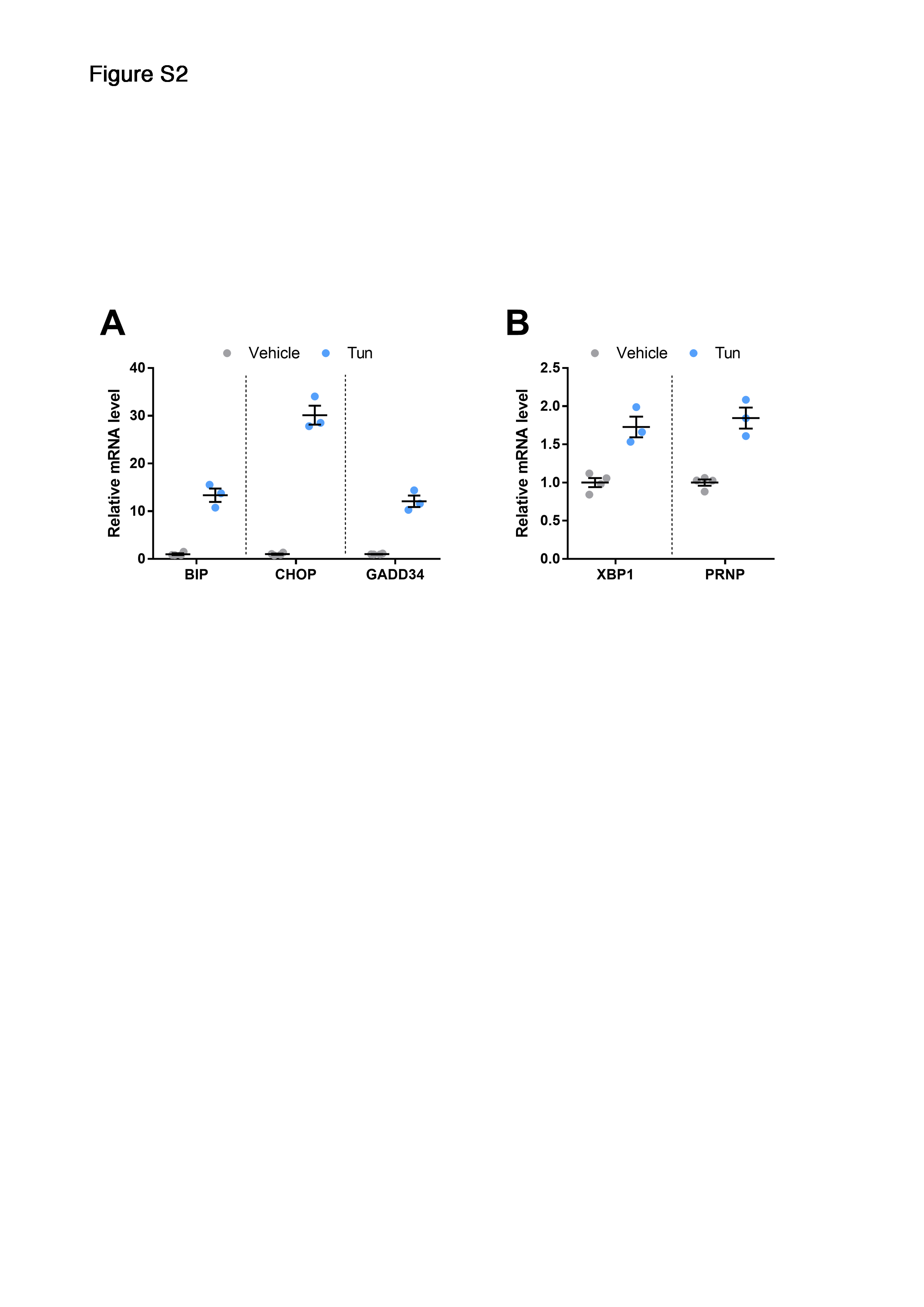

Supplement: Supplementary file 4 — S2 [file 41419_2020_2430_MOESM4_ESM.png]

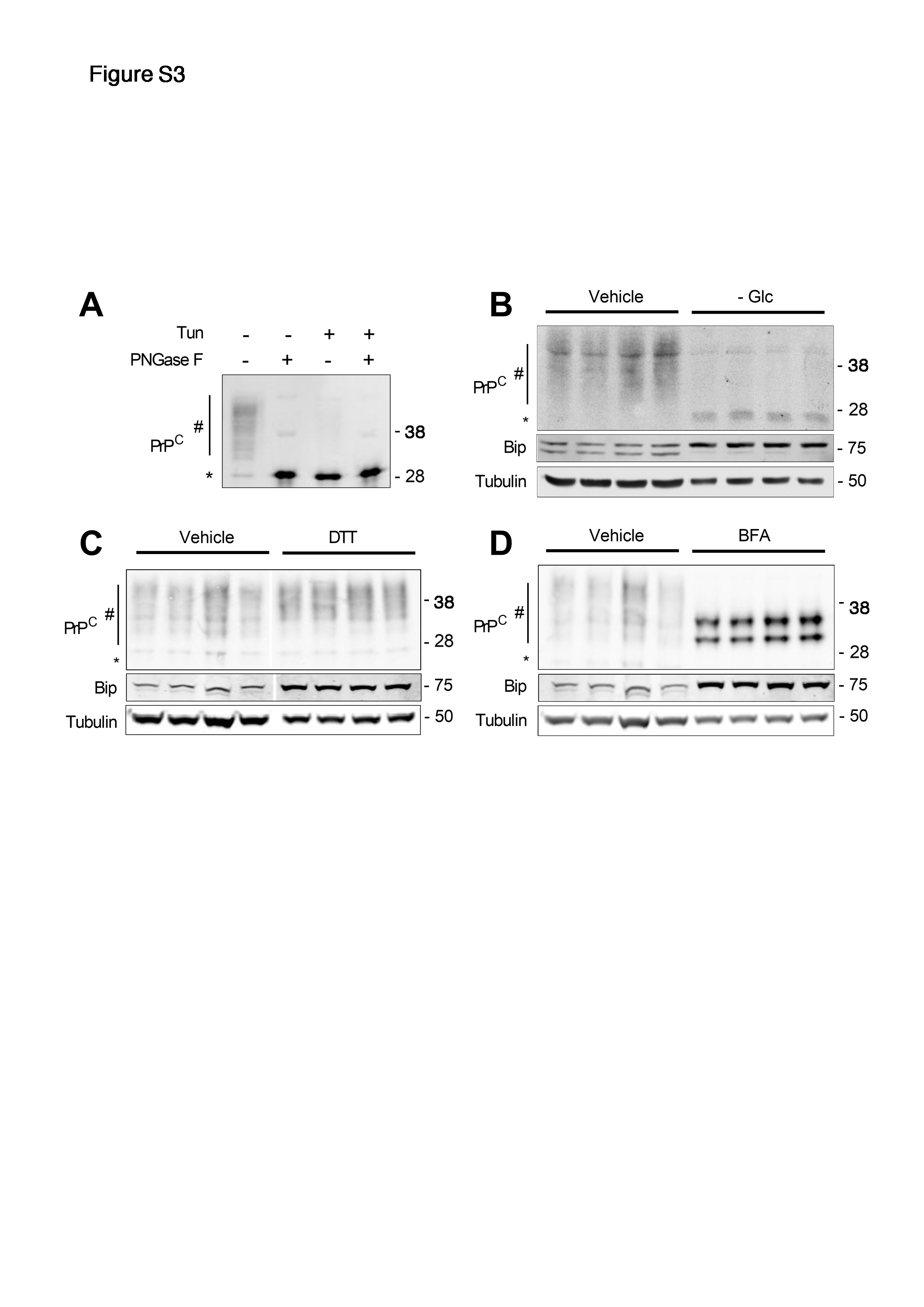

Supplement: Supplementary file 5 — S3 [file 41419_2020_2430_MOESM5_ESM.png]

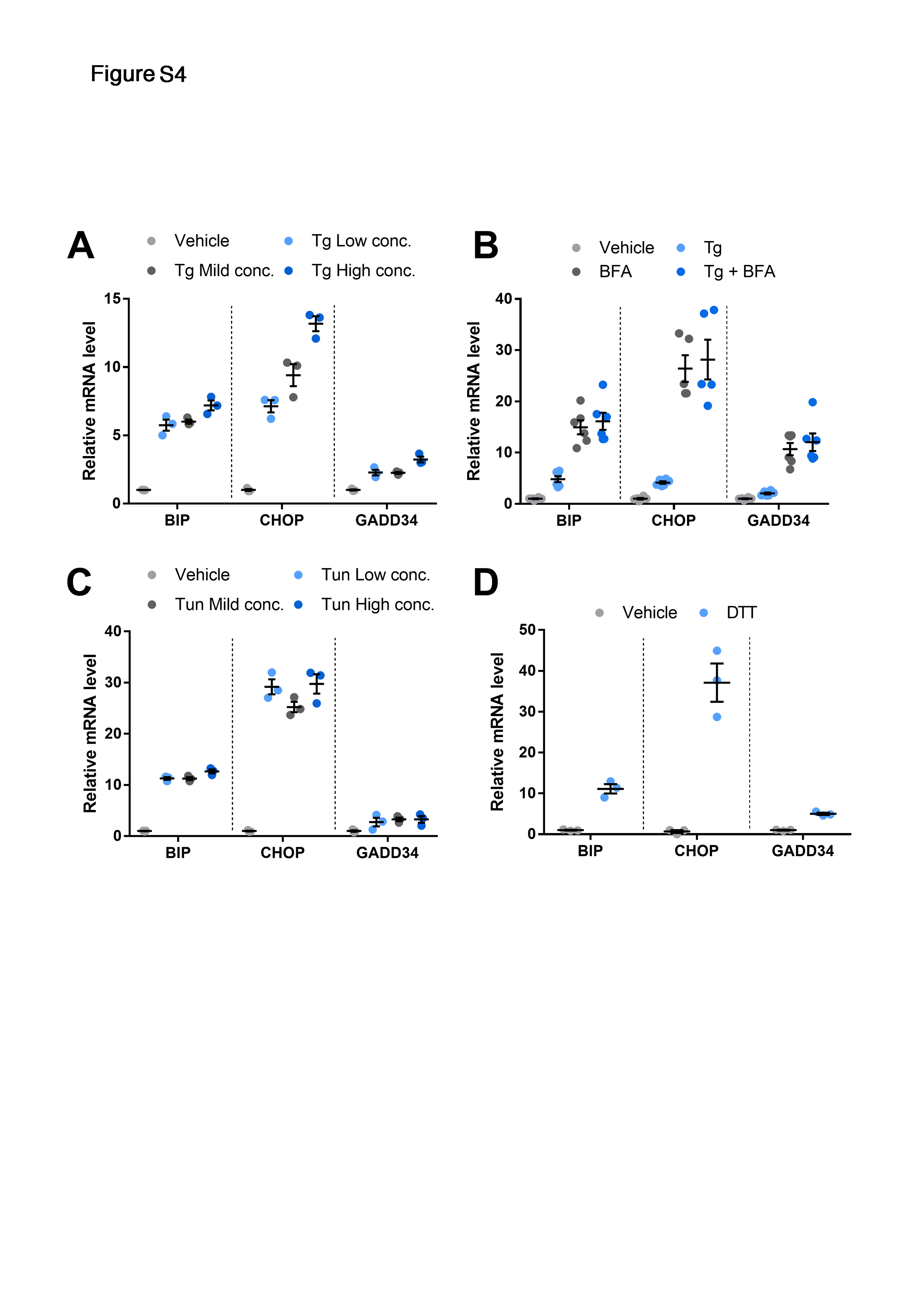

Supplement: Supplementary file 6 — S4 [file 41419_2020_2430_MOESM6_ESM.png]

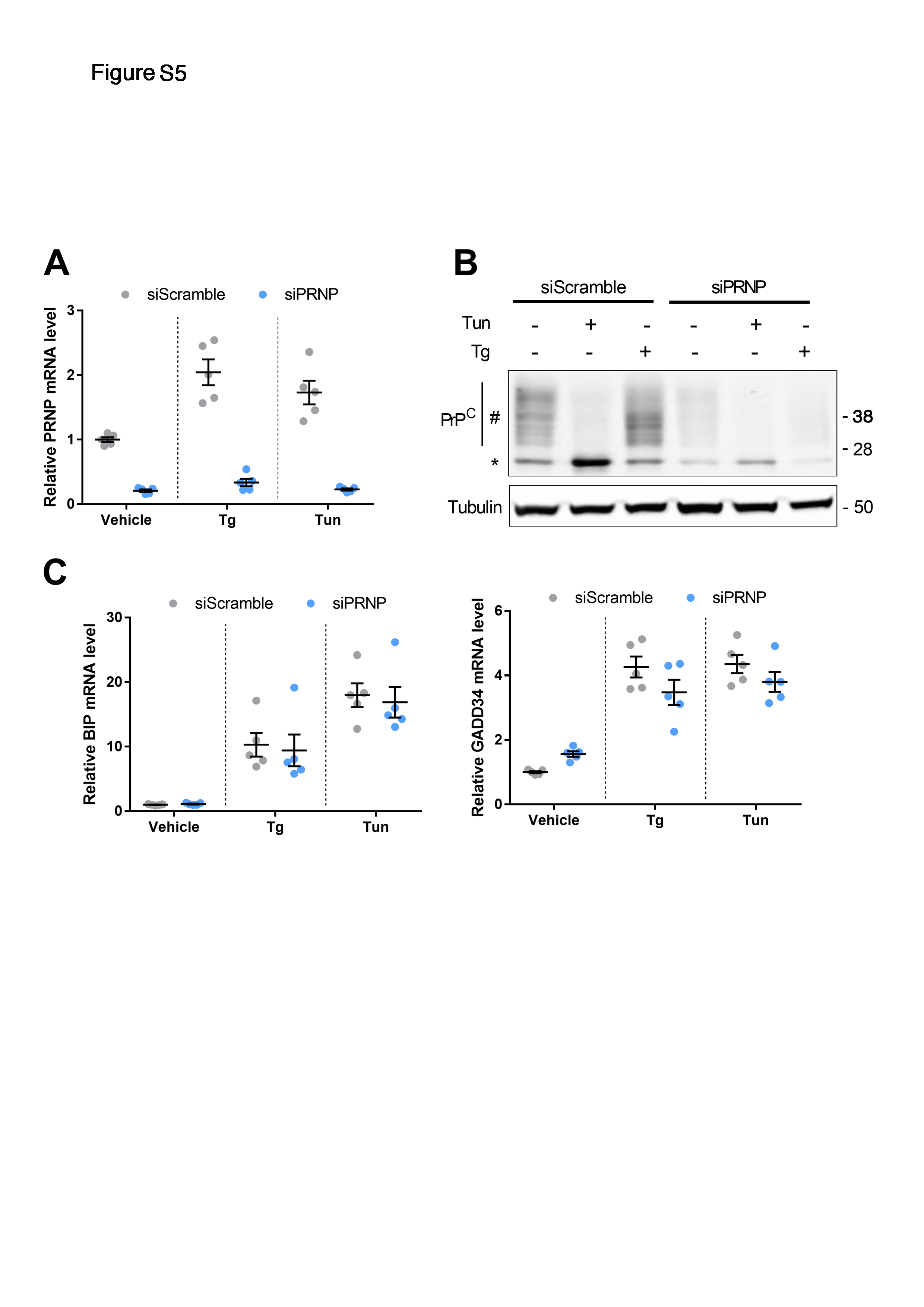

Supplement: Supplementary file 7 — S5 [file 41419_2020_2430_MOESM7_ESM.png]

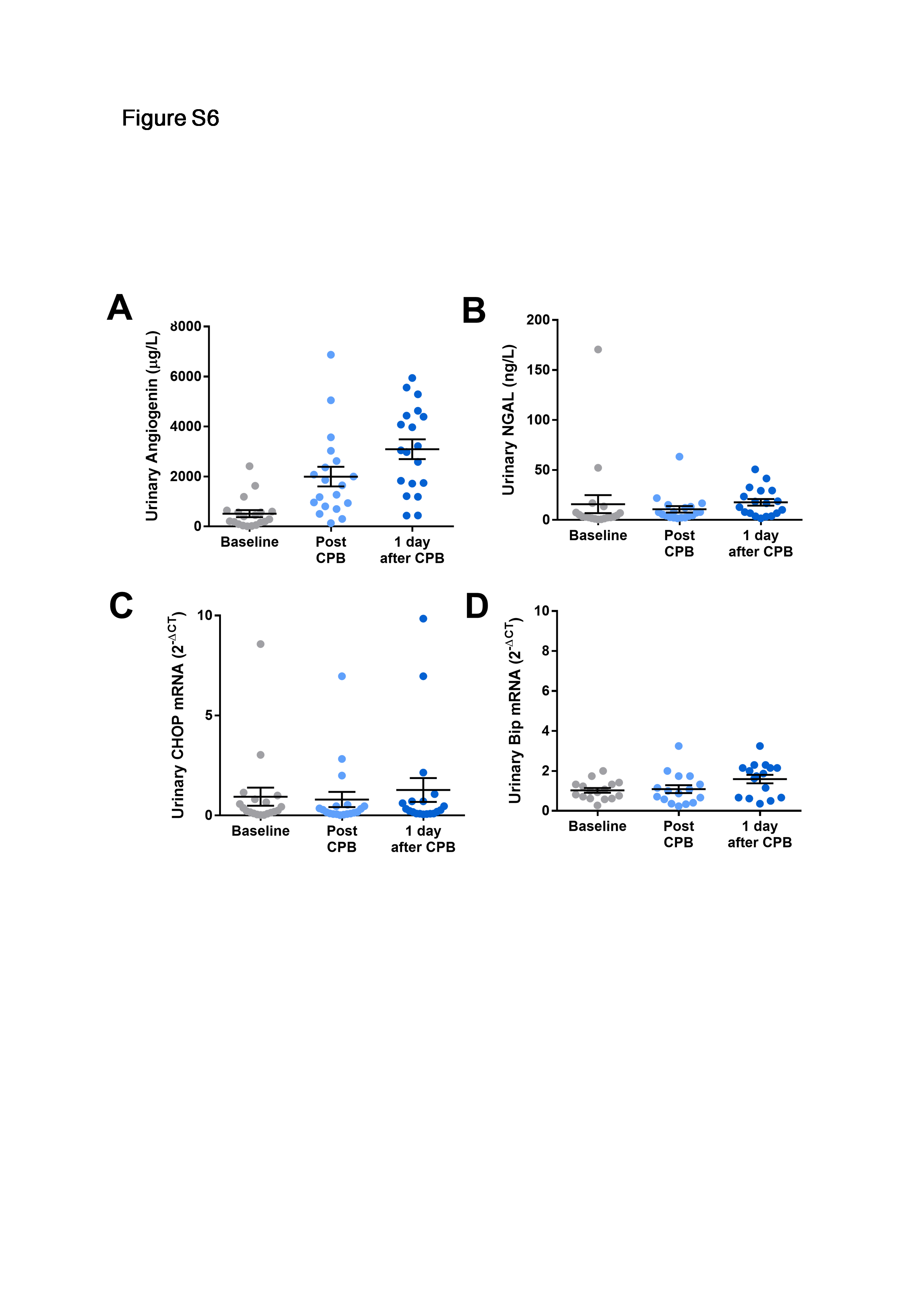

Supplement: Supplementary file 8 — R6 [file 41419_2020_2430_MOESM8_ESM.png]
